# Doing statistics, enacting the nation: The performative powers of categories

**DOI:** 10.1111/nana.12596

**Published:** 2020-02-16

**Authors:** Francisca Grommé, Stephan Scheel

**Affiliations:** ^1^ Department of Sociology Goldsmiths, University of London London UK; ^2^ Department of Sociology University of Duisburg‐Essen Duisburg Germany

**Keywords:** categories, Estonia, identity politics, national identity, Netherlands, performativity, statistics

## Abstract

It has been widely acknowledged in debates about nationalism and ethnicity that identity categories used for classifying people along the lines of culture, race, and ethnicity help to enact, that is, bring into being, the collective identities they name. However, we know little about how categories acquire their performative powers. The contribution of this paper is twofold: first, it proposes a conceptual framework based on concepts and insights from science and technology studies for investigating the performative powers of statistical identity categories and possibly also other domains. Second, it demonstrates, through an empirical study of two examples from Estonian and Dutch official population statistics, that statistical identity categories enact more than the groups to which they refer. We argue that they also enact national identities and notions of national belonging of majoritarian groups in the host countries. Therefore, statistical identity categories can be used as analytical lenses to study nationalism and processes of nation‐building.

## 1 INTRODUCTION

The performative powers and effects of identity categories aiming to group people along the lines of their origin or alleged national or cultural identity have long been acknowledged in the study of ethnicity, race, and nationalism. In his seminal article *Ethnicity without Groups*, Rogers Brubaker observes that categories invoked by “ethnopolitical entrepreneurs” are designed to “stir, summon, justify, mobilise, kindle, and energize” (2002, p. 166).^1^ He cites the work of Pierre Bourdieu to underscore the “performative character” (*ibid.*) of categories in processes of group formation. Bourdieu already noted a decade earlier that categories used to name groups along lines of ethnic origin or cultural background “may contribute to producing what they apparently describe or designate” (1991, p. 220). The crucial lesson that Brubaker draws from the performative character of categories is that scholars of ethnicity, race, and nationalism “should not uncritically adopt categories of ethnopolitical practice as […] categories of social analysis” (2002, p. 166). This would entertain a *groupism* that reifies the performativity of categories by construing “ethnically, racially, and nationally named populations” as bounded, homogeneous groups in order to treat “groups and nations as […] chief protagonists of social conflicts and fundamental units of social analysis” (Brubaker, 2009, p. 28).

Brubaker has made numerous propositions on how to move beyond this epistemological bias. First, he recommends to study “ethnicization, racialization, and nationalization as political, social, cultural, and psychological processes” instead of treating of ethnicity, race, and nation as pre‐existing, bounded entities (Brubaker, 2002, p. 167). Second, Brubaker invites scholars to distinguish between categories and the groups of people they name in order to “study the politics of categories” and how categories feature in processes of group formation (*ibid.*, p. 169–170). This implies to make identity categories an object of analysis, rather than a tool of analysis (Brubaker, 2013, p. 6).

Although these propositions have proven to be of uttermost value to transcend groupism in the study of ethnicity, race, and nationalism, neither Brubaker nor those who followed his groundwork have investigated any further how categories acquire their performative power. This is peculiar because the idea that categories help constitute the groups of people that they name prompted Brubaker to formulate his critique of groupism in the first place. We believe, however, that any study of the politics of categories remains incomplete as long as it cannot explain how identity categories acquire their performative power in order to investigate what it actually is that particular categories come to enact and how. The first contribution of this article, therefore, is a conceptual framework that, based on approaches from Science and Technology Studies (STS), permits to theorise and study performative qualities and effects of identity categories.

To account for the latter is of particular relevance at the current conjuncture in which questions of race, ethnicity, and national identity return to the forefront of public debate and policymaking (Blum & Guérin‐Pace, 2008; Escafre‐Dublet & Simon, 2012; Jääts, 2014; Kertzer & Arel, 2002; Simon, 2012). This resurgence is highlighted, among others, by the growing use of origin‐based statistical categorisations for immigrants and minorities in the European Union, which are the focus of our study. Relevantly, most national statistical institutes (NSIs) in the European Union do not collect data about ethnicity and race (with the exception of the United Kingdom and Ireland) but use country or place of origin as approximations. As we further explain in the theory section, statistical identity categories are part of a larger group of practices (e.g., bureaucratic or scientific) involved in performances of origin (national, ethnic, and otherwise). Furthermore, statistical identity categories are interrelated with categories from everyday usage and public debates. Yet we believe official population statistics offer a particularly suitable field for studying the performative powers of categories because the power of naming groups into existence is particularly pronounced when it is combined with the authority of officially certified numerical facts (on the latter point, see Ruppert & Scheel, 2019).

We illustrate the move towards origin‐based identity categories through two examples from the Netherlands and Estonia to show, and this is the second contribution of this article, that statistical identity categories used for the classification and quantification of migrants and minorities can be used as analytical lenses to study the enactment of national identities and notions of belonging regarding the majoritarian groups in the host countries.

In brief, the conceptual framework we propose draws on work in STS on the “double social life of method” (Law, Ruppert, & Savage, 2011). We demonstrate the analytical potential of our framework by studying two identity categories in‐the‐making in NSIs: the “third generation migrant” and the “Caribbean Netherlands origin groups.” We show that the performativity of categories is not reducible to the discourses and politics of ethnic entrepreneurs. Rather, categories help to bring into being the social realities they allegedly only describe because they are *of* the social. In brief, categories will only generate performative effects (a) if they are (or become) sedimented in sociomaterial method assemblages like statistical infrastructures or population taxonomies and (b) if they have certain advocates who promote and use them. We propose to study these method assemblages to understand how statistical identity categories carry specific histories, political agendas, and imaginaries that become part of and facilitate the performance of particular social realities.

Our study of categories draws on collaborative ethnographic research conducted in the context of a research project on methodological changes in European population statistics in 2015 and 2016. During this period, one of the authors conducted research at Statistics Estonia (SE), and the other at Statistics Netherlands (SN).^2^ Our methods comprised interviewing statisticians and observing meetings, everyday work, and software demonstrations. We decided to combine our findings when we realised that in both cases, the categories used by NSIs are related to narrated national histories of occupation and colonisation, albeit with one important difference: the category of the “third generation migrant” concerns the offspring of Russian‐speaking inhabitants of Estonia, a group of people associated with the Soviet occupation of the Estonian nation state (in this case, the former “occupiers” are construed as migrants). The Caribbean Netherlands origin categories are, in contrast, rooted in narratives of the Dutch colonial state (those living in colonised territories are construed as migrants). By combining these categories in our analysis, we do not seek to conduct a comparison of the two cases. Rather, we discuss the two contrasting cases together to demonstrate that the performative powers of statistical identity categories are not isolated phenomena. Our analysis shows that, notwithstanding the differences between them, both statistical identity categories enact notions of national origin rooted in histories of imperialism and related migration regimes.

In sum, our analysis permits us to show that categories used for labelling and quantifying migrants and minorities help to enact more than the groups of people that they name. As a consequence of how categories are entangled in sociomaterial assemblages, they also help to bring into being “collateral realities,” that is, “realities that get done incidentally, […] along the way, [… and] for the most part, unintentionally” (Law, 2012, p. 156). The collateral realities we examine are articulations of Estonian and Dutch national identities. We therefore conclude that scholars can use statistical identity categories as analytical lenses to study nationalisms and processes of nation building.

We develop this argument in three sections. First, we elaborate our STS‐inspired conceptual framework for theorising and studying the performative powers of categories. Subsequently, we mobilise this framework in the second and third sections to investigate the performative effects of statistical identity categories used by SE and SN in order showcase the analytical potential of our framework for scholars of nationalism and ethnicity.

## 2 NATIONALISM AND THE PERFORMATIVE POWERS OF IDENTITY CATEGORIES

Identity categories form an important part of what the anthropologist Orvar Löfgren has called an “international cultural grammar of nationhood,” understood as “a thesaurus of general ideas about the cultural ingredients needed to form a nation” (1991, p. 114). Identity categories operate as important devices of boundary making and markers of “national,” territorially bounded communities. Hence, studies in nationalism agree that identity categories in various practices, such as national museums, literature, science, and official statistics, play a central role in processes of nation‐building (Anderson, 2006; Brubaker, 2009; Elrick & Schwartzman, 2015; Gellner & Smith, 1996; Loveman, 2014; Wimmer, 2013). This central role of categories in processes of nation‐building resides in the conception of nationhood in terms of an *ethnos*, that is, as a community with a shared ancestry, culture, and history (Smith, 2000), a feature that often coexists with a civic understanding of nationhood in terms of a *demos* (Balibar, 2004; Brubaker, 1999).^3^

Our interest lies in how identity categories used to label immigrant and minority groups in official statistics also constitute “national populations” as distinct entities. The force of official statistics in doing this resides in what Morgane Labbé (2000) calls “statistical realism': the belief that statistics, if done properly, is a “pure science” devoid of any political considerations (Urla, 1993). Within the register of statistical realism, the objects to be enumerated are thought of as existing prior to and independently of statistical practices. Official statistics (and their usage) have therefore been complicit in normalising and authorising what are essentially politically contestable classifications (Andersen, 2008; Dave, 2004; Loveman, 2014). As noted above, they do so in combination with other practices, such as national museums that chronicle independent nationhood as the accomplishment of a particular ethnic group (Anderson, 2006) or scientific practices such as genetics, in which nationally bounded populations are produced by tracing national genetic specificities (Tupasela & Tamminen, 2015).

The foregoing is acknowledged by statisticians, social scientists, and other experts in the field (Desrosières, 2001). Social constructivist studies of censuses and other statistical practices highlight that ethnic and racial classifications are the result of the discussions and negotiations of a multiplicity of actors vying “over that most basic of powers, the power to name, to categorise, and thus to create social reality” (Kertzer & Arel, 2002, p. 36; Starr, 1992; Yanow, 2003). Category names already exist in social repertoires, yet statistical categories formalise, restructure, and organise everyday experience and, therefore, are complicit in generating social realities. This is why “[…] statistical categorizations both reflect and affect the structural divisions of societies” (Simon, 2012, p. 1368). The dynamics of quantification play an important role in the constitution of “imagined communities,” as Benedict Anderson (1991) emphasises in his seminal study. Dvora Yanow aptly summarises this as follows: “Naming a category asserts its importance; counting its members further underscores this” (2003, p. 11).

The most influential critique of identity categories starts from the observation that statistical identity categories are variable and contingent as they change over space and time (Jenkins, 1994; Kertzer & Arel, 2002; Yanow, 2003). From this, it follows that ethnic and racial categorisation schemes are, essentially, “human inventions, created to *impose* some sense of order on the *surrounding* social world” (Yanow, 2003, p. vii; emphasis added). Likewise, Mara Loveman regards ethnic and “racial categorization schemes as cultural *impositions* on human diversity, not merely descriptive of that diversity” (2014, p. 14; emphasis added). Although these criticisms point out crucial issues, they remain within the register of statistical realism: They assume “the social world” as an external reality existing independently of the categories and enumeration practices mobilised to classify and quantify it. They often leave open the possibility of an adequate categorisation scheme that could capture and do justice to the immense human diversity “out there.” Hence, these criticisms implicitly confirm the very assumption they seek to abandon, namely, that “identities can be reduced to an essential core within each individual, a core that exists outside of politics” (Kertzer & Arel, 2002, p. 19).

A second issue we put forward here is that statistical identity categories are not reducible to “inventions” or “fabrications” of wilful human subjects. Categories are part of vast assemblages that comprise both human and non‐human elements ranging from representatives of various institutions and professions competing for influence to material infrastructures like information systems and inscription devices. To appreciate the performative powers as well as the specificities and limitations of categories, we need to appreciate the material–semiotic practices, assemblages, and infrastructures they are part of (Bowker & Star, 1999).

In line with these insights, we propose a conceptualisation of categories as elements of method assemblages to account for the constitutive and performative work they do. This understanding of categories takes its cue from STS scholarship on the “double social life of method,” a body of work that demonstrates how methods of the social sciences bring into being the social worlds that these methods try to study (Law & Urry, 2004; Lury & Wakeford, 2012; Savage, 2010). For us, an assemblage refers to a heterogeneous collection of theories, assumptions, classification systems, research materials and tools, routines, and persons with specific expertise (Ruppert, 2012). The various elements of an assemblage form loosely patterned arrangements to reproduce categorisations, yet the exact composition of an assemblage may vary with sites of usage and over time. How different elements form an assemblage is relevant for what account of the world it makes present, in our case, for how categories make present national populations (Law, 2004). The ontology behind this reasoning is relational, which means that entities (such as an identity category) are never settled and pre‐existing but only exist as long as they are enacted again and again in a web of relational practices (Mol, 2002).

It follows that, to understand how statistical categories enact certain social realities, we need to take into account a set of pre‐existing social and material realities (Law, 2004, p. 13), such as already established, socially accepted practices of naming and identifying with particular social groups. Assemblages that cannot rely on the support of a set of established theories and statements, inscription devices, authorised communities of practice, institutionalised forms of expertise, and so forth will not be successful in enacting the realities that they describe, name, classify, count, or enumerate (Law, 2009). The relations between techniques, theories, and outcomes are not natural or self‐evident, but the outcomes of processes of mobilising, interesting, convincing, and repositioning various actors (Latour, 1993; Latour & Woolgar, 1986). This is indeed where categories are “of the social” in that they have advocates struggling for certain accounts and enactments of the world. The constituent, performative power of categories thus resides in a series of assumptions users of any method need to make about the character of the social world.

The assumptions through which identity categories can enact more than the groups of people they refer to are ingrained in these sedimented practices. Although these assumptions are often not made explicit, they operate, nevertheless, as self‐fulfilling prophecies that bring into being what John Law calls “collateral realities” (Law, Ruppert, and Savage, 2011). As noted in the Introduction, “collateral realities are realities that get done incidentally and along the way” (Law, 2012, p. 156). For example, the Eurobarometer survey measures attitudes on a particular issue (its main aim), but also enacts—through a method in which a sample functions as a stand‐in for a larger entity—a “European public” as a collateral reality (Law, 2009). This example highlights that “collateral realities,” just because they get done “incidentally,” are not necessarily realities and are negligible or of minor importance. This is also illustrated by the performative overflow of categories, which suggests that identity categories do not just help “to construct and constitute the groups they ostensibly describe” (Brubaker, 2009, p. 33). They also help to enact—as a collateral reality—the national identity of the supposed host country. This is because identity categories used for migrants and minorities can enact the “other,” thus “marking negatively what ‘we' are not” (Honig, 2001, p. 3; cf. Said, 2003; Wekker, 2016). Hence, such categories also carry more or less tacit assumptions about “the nation” and national belonging as the migrants and minorities; these categories refer to function as mirrors in which a national community is imagined and narrated into being.^4^

However, it is important to note that our approach does not account for how statistical categories come to circulate and how they are identified with. This would require us to study the “double social process” (Ruppert, 2012) in which “names interact with the named” (Hacking, 2007, p. 294; cf. Bowker & Star, 1999; Loveman, 2014). Whereas Ian Hacking (2007) primarily locates the performative powers of categories in these feedback loops, a similar dynamic has been theorised by self‐categorisation theory in social psychology (e.g., Turner and Reynolds, 2012). The categorisations we investigate in this article are, however, not based on self‐identification (for instance by self‐assignment to a set of pregiven categories in a census questionnaire). Instead, they are assigned to individuals on the basis of register data held by authorities, as we explain in detail below. Hence, the categories under consideration in this article allow us, precisely because they are assigned, to show that the performative powers of categories are not reducible to “interactions between names and people” (Hacking, 2007, p. 295). Rather, the performative powers also reside in national narratives and imaginaries as well as tropes about “self” and “other” that are carried by identity categories in the form of often tacit assumptions, which operate as self‐fulfilling prophecies. In this way, official narratives and everyday discourses about national identity and nationhood are taken up, reified, and amplified by official statistical categories.

To illustrate the analytical potential of our approach, we draw on Nira Yuval‐Davis' (2007) distinction between ethnic, cultural, and civic nationalism. Yuval‐Davis' differentiation is useful to discern emphases, shifts, and trends in the enactment of national identities. These can be built around a “myth of a common origin or shared blood/genes” (ethnic nationalism), a shared “symbolic heritage provided by language and/or religion and/or other customs and traditions” (cultural nationalism), or they can emphasise the shared rights and duties of the community of citizens, thus relating “the boundaries of the nation […] directly to notions of state sovereignty and specific territoriality” (civic nationalism; Yuval‐Davis, 2007, p. 21). However, as our cases will point out, in practice, national identities are enacted with reference to complex combinations of cultural and ethnic elements (cf. Malkki, 1992; Schinkel, 2013; Van Reekum, 2012).

Such insights are especially relevant in the current conjecture in which statistical identity categories become increasingly intertwined with policies and debates on migration in large parts of Europe (Elrick & Schwartzman, 2015; Escafre‐Dublet & Simon, 2012; Simon, 2012). However, notwithstanding the growing salience of claims to origin, historical essence, or authenticity, the nature of the essence itself typically remains elusive when political advocates attempt to define it (Geschiere, 2009). As we show below, within this ongoing and diffuse process of distinguishing origin, the statistical categories in this study enable *specific* performances of national identity by enacting stark boundaries between “native” residents and “foreign” migrant others.

## 3 THE THIRD GENERATION IMMIGRANT: ENACTING ESTONIA AS AN ETHNIC NATION

Since December 2015, one can retrieve data on a new category of people from SE's statistical database: the “third generation of the foreign‐origin population.” In December 2015, two statisticians were busy with calculating tables on this new category of people in relation to various characteristics like sex, age, spatial distribution in Estonia by county, educational background, and unemployment rates (interview SE, December 2015). These tables were uploaded to the new “integration indicator database,” which is meant to provide “a single information point for finding and monitoring data on integration of different ethnic groups in Estonian society.”^5^

The official title of the new category of people is rather bulky. It reads “third generation of the foreign‐origin population” (hereafter: “third generation”). Although this category appears as a neutral denominator of the “foreign origin population” free of any distinctions along lines of race or ethnicity, the category refers, in fact, to the offspring of Estonia's Russian‐speaking inhabitants, which account, according to official statistics, for up to one third of Estonia's population (Poleshchuk, 2009; Tammur, 2017; Vetik, 2011).

**Figure 1:**
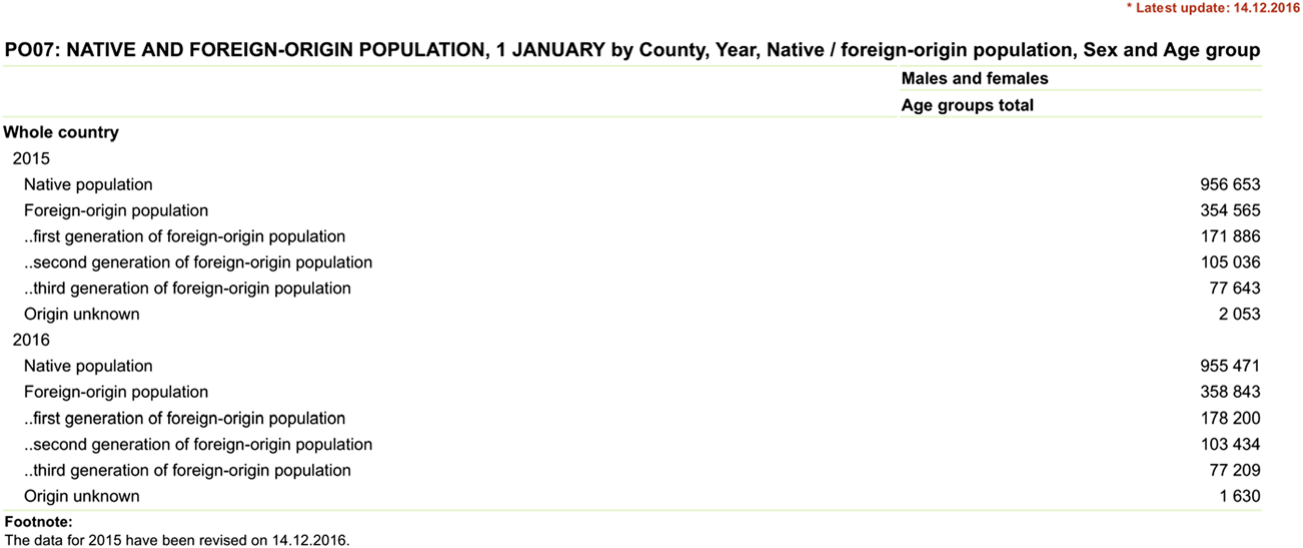
Statistics Estonia table of the “Native and foreign‐origin population”, 1 January 2015 and 2016 Source: Statistics Estonia^6^ [Color figure can be viewed at wileyonlinelibrary.com]

It is only possible to retrieve information on the “third generation of the foreign origin population” from SE's homepage from the year 2012 onwards (see Figure 1). The reason is simple: The construction of this category of people relies on data that has not been collected before the last population and housing census (PHC) in 2011. In the PHC 2011, it was decided to include an additional question in the census questionnaire, which inquired about the place of birth of the grandparents (interview SE, May 2015). This information is crucial to determine whether an individual is part of the “third generation.” The importance of ancestry is reflected by the official definition of the “third generation” as any person “permanently living in Estonia of whose parents at least one was born in Estonia but whose grandparents were all born abroad.”^7^

The importance of ancestry in the definition of the third generation highlights that this statistical category carries a particular historical narrative about the Estonian nation state. This historical narrative becomes apparent if one considers that the definition of the “third generation” features Estonia as a spatial reference point for the place of birth of the parents and the grandparents. The narrative is peculiar insofar as Estonia did—de facto—not exist as an independent nation state when most of the parents and grandparents of the people labelled as “third generation” were born. Rather, the territory of what is today known as Estonia was part of the Soviet Union between 1939 and 1991. The category of the “third generation migrant” thus enacts a crucial element of the official historical narrative of Estonia; although the Estonian nation state did not exist *de facto* during the Soviet period, it never ceased to exist *de jure*, leading a virtual existence of legal continuity during a period officially known as “occupation” that lasted more than 60 years.^8^

Hence, the category of the third generation enacts much more than the people it names: it carries a particular version of the history of the Estonian nation, a history imagined in terms of both (de jure) legal continuity and (de facto) *rupture* of Estonian nationhood. The notion of the rupture “stands for the interruption and deterioration of the harmonious national development [of the Estonian nation‐state] of the pre‐war independence era” (Jõesalu & Kõresaar, 2013, p. 183). This is the dominant script in re‐independent Estonia for interpreting the Soviet era, which is remembered and narrated as a brutal occupation characterised by violent repression, ideological pressure, and political persecution (*ibid.*, p. 184). Importantly, the communist regime of the Soviet period is framed as the occupation by an external force that is construed as foreign in both ideological and ethnic terms (Troebst, 2006, p. 79–80). In this way, the script of the rupture “has developed a strong ethnic and national repertoire [… that] differentiates [among the inhabitants of re‐independent Estonia] between the carriers of ‘our own' national history (the Estonian middle class and farmers) and the carriers of ‘alien' history (communists and Russians)” (Jõesalu & Kõresaar, 2013, p. 184). This stark distinction along ethnic lines is carried by the category of the “third generation” and facilitates the enactment of people allocated to this category as of “foreign origin,” despite the fact that people defines as such were born and have grown up in re‐independent Estonia.

At first glance, the script of de facto interruption of Estonian nationhood seems to be at odds with the insistence on de jure legal continuity of the pre‐war Estonian Republic (Vetik, 2011). The latter is enshrined in the first paragraph of the constitution, which claims that “the independence and sovereignty of Estonia are timeless and inalienable” (Constitution of the Republic of Estonia. English Translation, 1992, Section 1). Yet claiming legal continuity is actually the logical consequence of the script of the rupture that vehemently disavows the Soviet experience as the occupation, if not colonisation, of the Estonian nation state by an ideological and ethnical alien force. The idea of legal continuity is mobilised to re‐establish the alleged ethnical purity of the Estonian nation after the interruption of national development by Soviet occupation has ended with Estonia's re‐independence. This is highlighted by the consequences of legal continuity for the offspring of the Russian‐speaking people who settled in Estonia during the Soviet period.

In brief, the concept of legal continuity enabled the passing of a citizenship law in 1992 that rendered most of the Russian‐speaking population stateless. The law stipulates that only people who were citizens before Estonia's incorporation into the Soviet Union in 1940 and their descendants were entitled to automatic citizenship (Fein & Straughn, 2014).^9^ Due to a combination of emigration and naturalisation procedures, the population's share of people with “undetermined citizenship”—the official term for stateless people in Estonia—has decreased from more than 30% in 1992 to about 7% in 2010 (Poleshchuk, 2013). However, the *Estonian Society Monitoring Report 2015* notes that as many as 34% of the second and 19% of the third generation born in Estonia are non‐citizens (Vetik et al., 2016).

Ancestry‐based identity categories like the “third generation” enact people who have been born and raised in Estonia as “foreign” by making the place of birth of the grandparents the central criterion for the determination of who belongs to the imagined community of the Estonian nation. In this way, the Russian‐speaking inhabitants of Estonia are enacted as immigrants despite the fact that none of them has ever crossed an international border: Whereas members of the third generation have been born in re‐independent Estonia, members of the second generation have mostly been born in a part of the Soviet Union that became Estonia in 1991. Members of the first generation were the ones who initially settled in that corner of the Soviet Union, mostly during the 1960s and 1970s. By making ancestry the central criterion for national belonging, origin‐based categories like the “third generation” essentialise alleged cultural differences, enacting them as immutable. This was, in fact, the impetus driving the introduction of the category of the third generation. A demographer who has lobbied in the scientific council of the PHC 2011 to add a question on the grandparents' place of birth to the census questionnaire summarises the rationale behind the new identity category as follows: “You can change your mother tongue; you can decide to identify as Estonian; you can even change your citizenship. But you cannot change the place of birth or your grandparents!” (interview SE, May 2016).^10^

The demographer's reference to mother tongue and self‐identification with a particular nationality point to alternative statistical identity categories used in Estonian population statistics. These are the categories of *mother tongue* (first language) and *ethnic nationality*, which have been inherited from the Soviet period. Both categories played a crucial role in the state‐building of the Soviet Union as a multinational socialist federation (Hirsch, 1997, p. Hirsch, 2000). Importantly, both categories are based on self‐information and—in the case of nationality—on self‐identification with a particular national culture.^11^ The methodology of self‐information is, however, precisely the reason why these categories are dismissed as subjective by statisticians who contrast these “unreliable indicators” with the “objectivity” of information on the place of birth of the grandparents (interview SE, May 2015).

The crucial point is that the allegedly objective criteria of place of birth of the grandparents, and the ancestry‐based distinction between “native” and “foreign‐origin” population enabled by it, enacts Estonia as a decisively *ethnic* nation (Poleshchuk, 2009). Whether a person is considered to be member of the “foreign” (*välispäritolu*) or of the “native” (*pölis*) population, and thus as a member of the imagined national community of Estonia, depends not on her legal citizenship, her place of birth, her language capacities, or her self‐identification but on her “cultural background” (interview SE, December 2015).^12^ This cultural background is, however, essentialised as it is inferred from a person's “roots,” that is, the cultural background of the person's biological parents and grandparents, which is, in turn, territorialised as it is inferred from their respective place of birth.

This conception of the imagined community of Estonia enacts what Richard Alba calls a *bright boundary*, a boundary between a “native” and a “foreign” population that is unambiguous and difficult, if not impossible, to transgress because this boundary is drawn by an “objective” criterion that cannot be influenced by an individual: the parents' and grandparents' place of birth. Hence, belonging to the imagined community of Estonia becomes not a question of self‐identification, citizenship, or language faculty but a question of ancestry. This emphasis on ancestry enacts a form of nationalism that imagines the nation in terms of *ethnic purity* (cf. Kertzer & Arel, 2002). By declaring the place of birth of the parents and grandparents, the central criterion for the definition of the “native” population, national belonging, is fixed in a distant past that determines the (non‐)belonging of an individual to the imagined national collective in the present. Ultimately, belonging to the national community becomes an exclusive affair that is protected by an insurmountable hurdle of “ethnic origin.”

Statistical categories like the “third generation” accomplish this through a networked assemblage of advocates, classifications, and data collection methods that reflect conceptions of history already entrenched in the constitution and circulating in public debates. Hence, the third generation category does not perform bright boundaries on its own but relies on existing assemblages. The particular contribution of statistics to these processes is that statistical origin categories perform bright boundaries between native and foreign populations as self‐evident and clear‐cut, as we elaborate further for the case of the Caribbean Netherlands.

## 4 THE CARIBBEAN NETHERLANDS ORIGIN GROUPS: ENACTING THE NETHERLANDS AS A MODERN, PROGRESSIVE NATION

A statistician is checking online tables about the Netherlands residents' countries of origin, looking for the Caribbean Netherlands origin group (at least one parent born in the Caribbean Netherlands).

Can you come from Bonaire? No. From the Caribbean Netherlands? No. [he mumbles] People cannot come from the Caribbean Netherlands. Sorry, sometimes I do not understand the statistics made by SN [laughs]. [He finds the figures] This is possible from 2012, 10 people. But this is nonsense! It is not a new *country.* I am going to e‐mail someone about this, it is confusing … (interview SN, February 2015, emphasis by the author).

**Figure 2:**
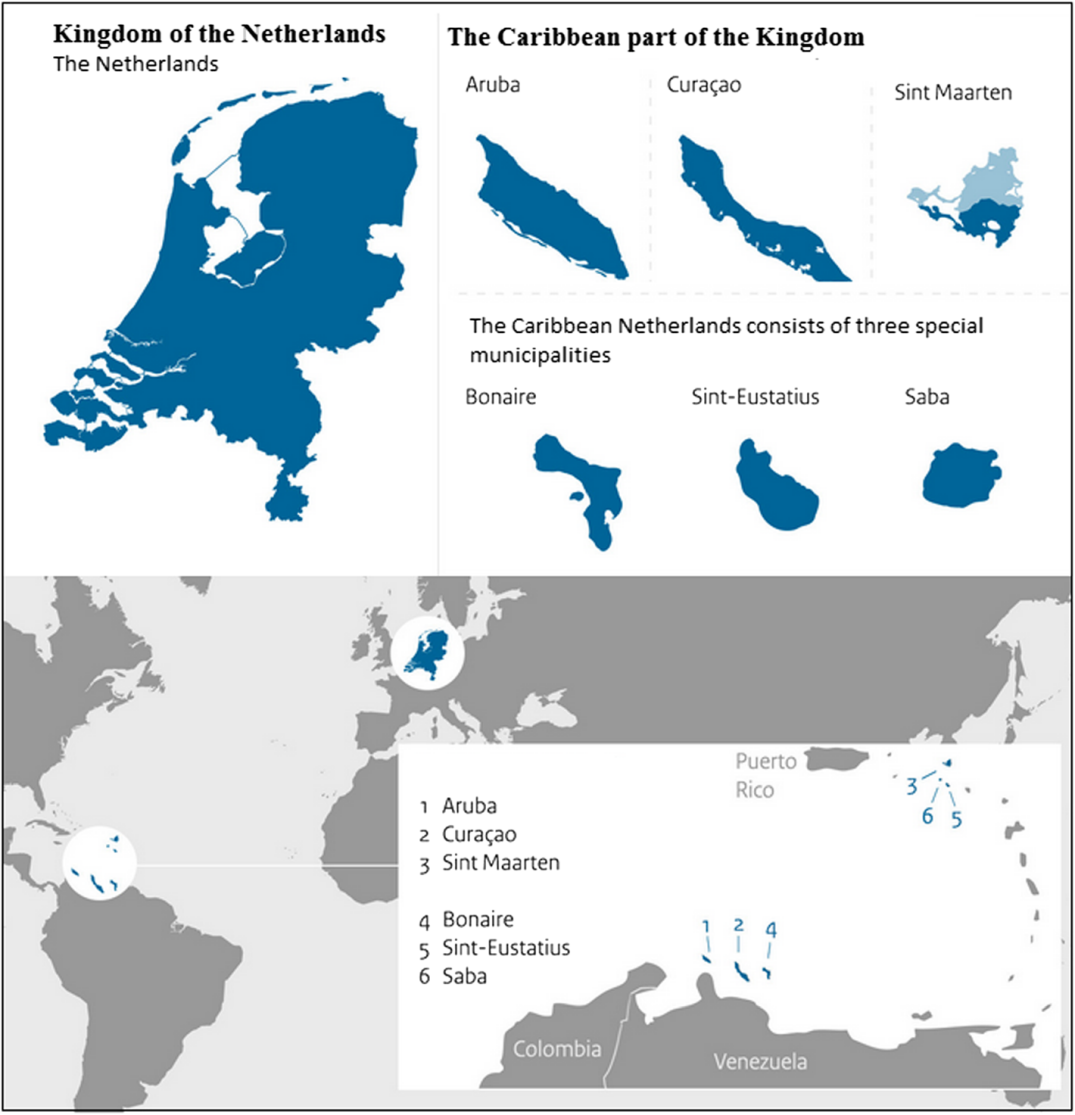
The Kingdom of the Netherlands: Continental and Caribbean [Color figure can be viewed at wileyonlinelibrary.com] Source: Netherlands Ministry of the Interior and Kingdom Relations (n.d.); adapted by the authors

**Figure 3:**
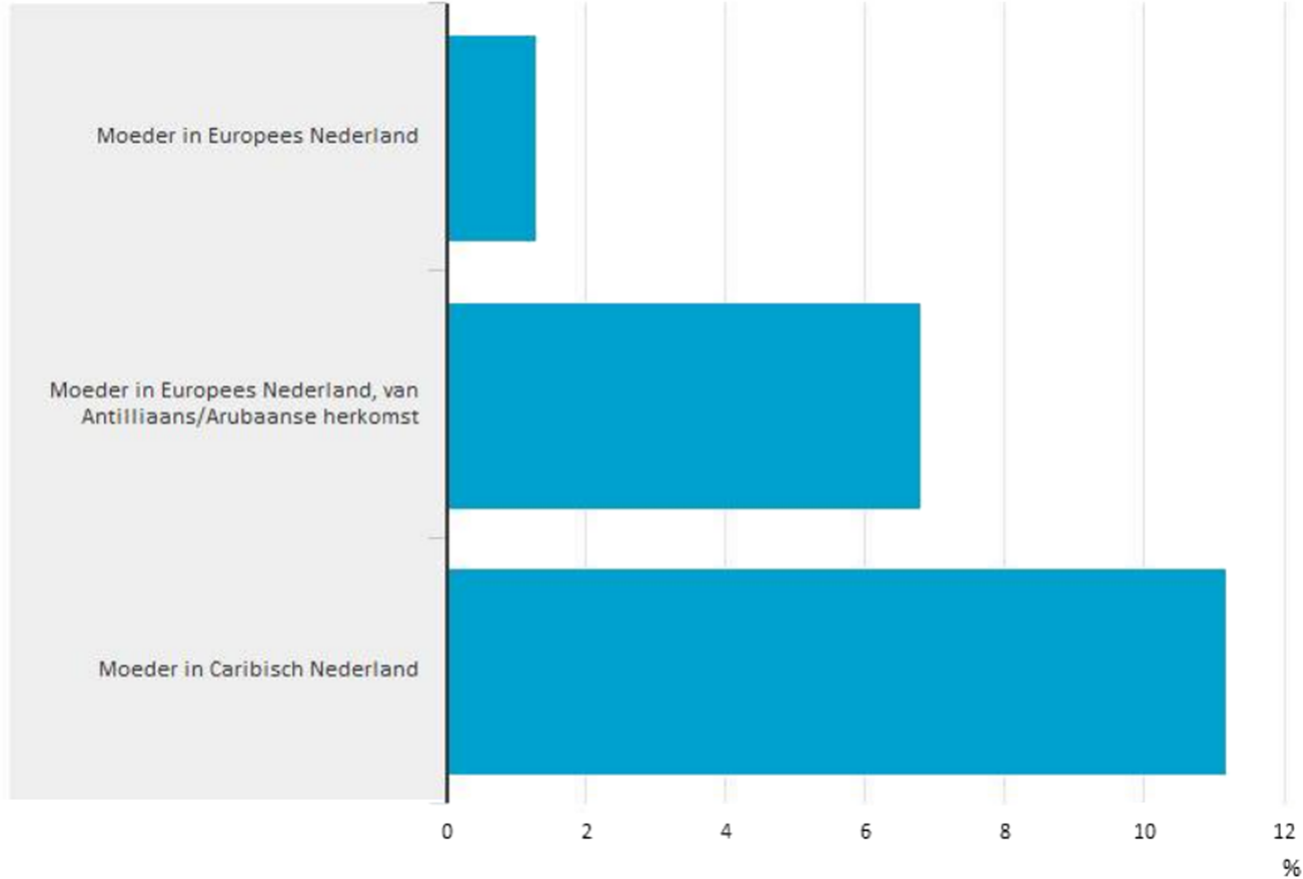
Statistics Netherlands table “Percentage of babies with teenage mothers, born between 2010 and 2013.” Source: Twitter Statistics Netherlands, 9 June 2015. Translation left axis, from top to bottom: “Mother in the European Netherlands”; “Mother in the European Netherlands, Antillean/Aruban origin”; and “Mother in the Caribbean Netherlands” [Color figure can be viewed at wileyonlinelibrary.com]

He is surprised because SN's website refers to the *Caribbean Netherlands*, the islands of Bonaire, Saint Eustatius and Saba, as a new country.^13^ Colonised in the 17th century, the islands gained *country* status in 1954 as part of the *Netherlands Antilles* (see Figures 2 and 3). The “status aparte,” as it is referred to, made the Netherlands Antilles a partly self‐governed entity within the Kingdom of the Netherlands (which also includes the continental Netherlands, see Figure 2). However, like many Caribbean countries, the islands are not following a linear path to full independence (Bonilla, 2015; Oostindie, 2006). After a period of rising government debts and poverty levels, Bonaire, Saint Eustatius, and Saba voted for closer ties with the continental Netherlands in 2006. Although not uncontested, the islands changed status in 2010 and became “special municipalities” of the continental Netherlands, partly independent administrative divisions (or “public bodies”) modelled along the lines of municipalities (Oostindie & Klinkers, 2012).

To refer to the Caribbean Netherlands as a country is a rather common mistake and therefore a telling one. In fact, the Caribbean Netherlands is a municipality with a country status for administrative purposes, including the registration of country of birth and place of residence in population registers. This situation originated in 1954, when goods, information technology systems, and personal records were given a distinct country code by the International Organisation for Standardisation. Although the islands of the Caribbean Netherlands lost their status as a part of a country after the 2010 vote, the political consensus was that the islands should retain their distinct and independent character as much as possible (Oostindie & Klinkers, 2012). These considerations, combined with political concerns about tracking migration to the continental Netherlands from the Caribbean and Latin America, were among the reasons to assign the Caribbean Netherlands its own International Organisation for Standardisation country code (BQ) despite its status as a special municipality. In terms of statistics, this means that people from the Caribbean Netherlands are categorised as foreign migrants (a practice we return to below), as SN's demographic statistics are based on population register data.

An assemblage of population registers, country codes, and political advocates thus helps to enact the Caribbean Netherlands origin categories. Embedded in this assemblage is the assumption that the Caribbean Netherlands form, from a statistical viewpoint, a foreign nation state. However, many experts, including statisticians at SN, are aware that the Caribbean Netherlands is a relatively new construct referring to three island states with large distances between them ( Van der Pijl & Guadeloupe, 2015). More commonly accepted is the collateral reality that this assumption enacts: the integrity of the territory of the *continental* Netherlands, which is enacted as unchanged and separate from the Caribbean islands despite the 2010 changes.

To learn more about how the Dutch nation state is enacted through the Caribbean Netherlands origin categories, we consider another element of this assemblage: the decades‐old practice of producing separate population statistics about the Caribbean Netherlands origin groups and its pre‐2010 predecessor category, the Netherlands Antilles (we include both in our analysis of the Caribbean Netherlands origin categories, see Table 1), for minority policies. Minority policies revolve around the notion of “integration”: the state needs to help migrants integrate into Dutch society, supported by statistics to monitor this process. We take the concepts and practices relating to integration measurements into account because they are part of the sedimented practices that shape identity categories and related imagined communities. In what follows, we trace two elements of this integration assemblage: the embedding of the Caribbean origin categories in the logic and taxonomies of “allochtonous adjustment” and the production of research that “fills in” the Caribbean origin categories.

**Table 1:** The Caribbean Netherlands origin categories^14^

Year	Name	State form	SN origin category
1954	Aruba, Curaçao, St Maarten, Bonaire, St Eustatius, and Saba	Country within the kingdom (“status aparte”)	(former) Netherlands Antilles origin category (born before 10 October 2010)
2010	Bonaire, St Eustatius, and Saba	Special municipalities	Caribbean Netherlands origin category (born after 10 October 2010)

The foreignness of the Caribbean part of the Kingdom is maintained by statisticians through the routine production of separate tables on topics such as life expectancy, birth rates, and place of residency. A statistician explains the rationale guiding this production as follows: “Basically this [producing separate tables] always concerns allochtonous people, first and second generation, at the recommendation of the Ministry of Social Affairs and the Ministry of the Interior” (interview SN, June 2016). By routinely distinguishing people with at least one parent born in another country as “allochtonous,” the production of standard demographic tables serves the government's aim of measuring their “adjustment” to Dutch norms and culture. Yet the exact nature of these norms and culture is still subject to heated debate and research. Part of the “Dutch origin” seems to be a vague and shifting core of progressive values, such as acceptance of gay rights and appreciation of freedom of speech (Duyvendak, Hurenkamp, & Tonkens, 2010; Van Reekum & Duyvendak, 2012).^15^

By including Caribbean origin categories in the “allochtonous” group, colloquially meaning “foreign” and literally meaning “not from the soil,” statistical practices fix culture to territory (Malkki, 1992). To understand this, it is necessary to briefly explore this term and its usage. Although the term was coined in 1971 by the Verweij Jonkers Institute as a supposedly objective term, it has by now acquired connotations of “lagging behind,” “being deviant,” and “being criminal” (Essed & Kwame, 2006). It is construed as the opposite of “autochthonous,” which in Dutch official statistics is used to refer to people with two parents of Dutch origin. After officially defining and introducing the terminology in the 1990s, SN added a distinction between western and nonwestern in 2000: “If a group strongly resembles the Dutch population from a socio‐economic or cultural perspective, it will be considered western allochtonous” (Keij, 2000, p. 24). The Caribbean Netherlands (and former Netherlands Antilles) is part of the group of nonwestern countries, as are Turkey, all African countries, Latin America, and Asia, excluding Japan and Indonesia.

As part of an assemblage aimed at measuring integration, the use of this taxonomy has been criticised for inscribing country of origin categories with difference from the Dutch “norm,” thus constructing a hierarchy of geographically ranked cultures (Schinkel, 2013; Yanow & van der Haar, 2013). This classification of populations is based on the policy assumption that there are certain 'problematic groups' needing government intervention. Furthermore, the group of nonwestern allochtonous origin countries has become a “quick political‐bureaucratic term for black, Muslim or both” (Groenendijk, 2007, p. 105).

The negative connotations and divisive effects of this classification scheme have been acknowledged by a report of the Netherlands Scientific Council for Government Policy, which advised to abolish the terminology (Bovens, Meike, Jennissen, & Engbersen, 2016). Even though SN recognises the criticisms and adopted new terminology (see CBS, 2016a), the existing classification scheme is ingrained in research and policy practices as it is supported by another group of advocates invested in origin categories: integration researchers in government research agencies and academia (Essed & Kwame, 2006). As an SN statistician explains, changing the western and nonwestern groupings would interrupt the continuity of the statistics and thereby “inconvenience” integration researchers (interview SN, 2016). In line with these concerns, the classification is continued in the SN statistics portal (Statline) under the new label of “migration background,” indicating that practices are difficult to change because they are embedded in as assemblage of advocates and theories of society.^16^

To fully understand how the categories' embedding in this classification matters for the enactment of Dutch nationhood, we now turn to how this category is “filled in” by the production, interpretation, and publication of demographic statistics (Krebbekx, Spronk, & M'charek, 2017, p. 651). We suggest that these practices shape Caribbean origin categories as “culturally” laden and, by extension, perform the collateral reality of Dutch nationhood. Our review of the last 10 years of demographic publications by SN on persons of Caribbean Netherlands and Antillean origin shows that these exclusively concern the topics of urban residence, life expectancy, teenage motherhood, and single motherhood.^17^ When we asked about the latter two topics, a statistician responded “we just know that this [a higher ratio of teenage mothers] is the case in the Caribbean Netherlands” (SN statistician, 8 October 2015). When referring to the Caribbean Netherlands, statisticians habitually framed daily life in this region as culturally different from the continental Netherlands in terms of family life, composition of households, and motherhood. It thus appears that this “ethnic common sense” informs everyday statistical practice (as it is on other fields of policy‐oriented research), albeit as an essentialised notion of culture (Brubaker, 2002; Krebbekx, Spronk, and M'charek, 2017).

This production of difference through population statistics reiterates familiar colonial tropes, where gender and family norms are a core area of boundary work between “foreign” and “native” populations (Bonjour & De Hart, 2013; Van Reekum & van den Berg, 2015). Our point is *not* that there are no differences between family life as practiced in the continental and the Caribbean Netherlands. The example rather illustrates how statistical categories are “of the social”, as the Caribbean origin categories are “filled in” by statisticians' repeated choice to focus statistical production on the topics of motherhood and family relations.

Furthermore, the particular differences produced through this assemblage of registers, country codes, integration theories, and political and research advocates enact the Dutch nation according to an oriental logic. A striking example is teen pregnancy. The table shown above (see figure 3) compares teenage motherhood between autochthonous and allochtonous populations.^18^ SN points out that teenage pregnancy rates are highest among Antillean and Caribbean origin groups. Yet as a 2015 publication argues, “the number of teenage births among women from the Netherlands Antilles/Aruban origin in the Netherlands is gradually adjusting to all women in the Netherlands” (Loozen & Harmsen, 2015, p. 8). This is a second central assumption next to the assumption of territorial integrity mentioned earlier: there are fixed Dutch cultural traits to which other populations will adjust once living in the Netherlands. Again, this assumption enacts Dutch culture and norms through the invocation of colonial imagery. In this case, modest and controlled Dutch family life is enacted as the implicit ideal and counter image to the “precocious” practices of women from the former colonies (Stoler, 2010; Wekker, 2016).

In sum, our analysis shows that the Caribbean origin categories enact Dutch culture as marked by a western, progressive, and modern morality (Stoler, 2010). Although this repertoire is not limited to skin colour, it is specific to the “white” cultural archive (Wekker, 2016). Caribbean origin categories thus enact an essentialised notion of culture along lines of ethnicity and race (Abu‐Lughod, 1991; Schinkel, 2013). In light of this analysis, we suggest to refine—at least for the case of population statistics—the argument that one effect of Dutch integration research and its categories is to enact the Netherlands as nonethnic and as devoid of ethnicity‐related issues (cf. Schinkel, 2013; Yanow & van der Haar, 2013). Our analysis rather confirms Peter Geschiere's (2009) observation that origin categories promise a quick (but ultimately unfulfilled) solution to the conundrum of defining the meaning of Dutch “origin” by fixing culture to territory.

## 5 CONCLUSION

This article has proposed a conceptual framework that enables scholars of nationalism and ethnicity to theorise and study the performative powers of statistical identity categories, and possibly identity categories in other domains of knowledge production and political debate. Central to our argument is that identity categories used to label migrants and minorities help to enact more than the groups of people to which they refer. They can also help to enact, in the form of collateral realities, notions of national identity and belonging of majoritarian groups.

Based on this insight, we have shown through an analysis of statistical practices at two NSIs that identity categories used to label groups of migrants and minorities can be used as analytical entry points to study the articulation of particular forms of national belonging. Even though the categories we studied are part of different European histories of occupation, colonisation, and migration—and are therefore made up of different assemblages of advocates, data collection methods, and concepts—they are both origin‐based categories that enact essentialised notions of ethnicity and culture. This highlights the continued importance of ethnic nationalism (including notions of culture as a proxy for ethnicity) and the related enactment of bright boundaries between imagined (national) communities and their “others.” Both categories analysed by us also confirm and illustrate the observation of other studies that, in official statistics, migration is not only understood in terms of cross‐border mobility but also increasingly in origin‐based terms (Renard, 2018).

Importantly, the move towards origin‐based categories opens, maintains, or even deepens a disjuncture between the formal citizenry and the imagined (national) community, which often impacts negatively on the rights of alleged members of migrant and ethnic minorities (cf. Bonjour & De Hart, 2013). The latter are construed as deficient subjects in need of more and “better” integration, a conclusion that is mobilised to explain structural disadvantagement of these minorities and to justify the subjection of its members to a (potentially infinite) list of integration requirements.

To conclude, we draw two lessons from the performative powers of identity categories. The first lesson concerns the study of nationalism. In brief, acknowledging the performative qualities and effects of categories and the method assemblages of which they are part calls for enlarging the traditional field of study of scholars of nationalism and ethnicity. Rather than limiting their field of study to ethnopolitical entrepreneurs, official integration policies, or explicit symbols of nationalism, scholars may also attend to the sociomaterial assemblages and mundane practices that help to enact ethnic minorities and the national community as intelligible realities. As an analytical entry point, category assemblages provide a good starting point to observe the advocates, public demands, and policy preoccupations involved in the enactment of ethnic and national identities.

The second lesson concerns the ongoing debate about the use of identity categories in official statistics. In the context of this debate, acknowledging the performative qualities of identity categories opens up a more nuanced, situated position beyond a categorical rejection (e.g., Blum & Guérin‐Pace, 2008) or general endorsement (e.g., Escafre‐Dublet & Simon, 2012) of identity categories. In brief, our analysis indicates that the performative effects of particular identity categories depend on the assumptions on which they are based and how they are done in practice. In the case of Estonia, the third generation category enacts, for instance, bright, potentially insurmountable boundaries between Russian speakers and the imagined national community of Estonia by declaring ancestry the decisive criterion of national belonging. This contrasts with alternative categories such as *mother tongue* or *ethnic nationality*, which are based either on language faculty or self‐identification—criteria that can be changed by the individual concerned. Hence, acknowledging the performative qualities of identity categories opens up the prospect of a politics of categories that engages in the development and promotion of categories that are more fluid, permeable, and democratic than the origin‐based categories of ethnic nationalisms.

## ENDNOTES

1 In the following, we use the short‐form categories instead of identity categories to make the text more reader friendly. By identity categories, we mean categories used by various actors and stakeholders to assign people a collective identity, often defined along lines of cultural background, ethnicity, or origin (cf. Kertzer & Arel, 2002). Hence, “refugee” or “asylum seeker” do not resemble identity categories for us as they refer, first and foremost, to a particular legal status but not a sociocultural identity.

2 The data were collected as part of the ARITHMUS project in which a team of six researchers observed, interviewed, and studied documentation at five NSIs and two international organisations. The ARITHMUS team includes Evelyn Ruppert (PI), Baki Cakici, Francisca Grommé, Stephan Scheel, Ville Takala, and Funda Ustek‐Spilda (see ARITHMUS.eu). This article has benefited from the insights of all team members.

3 Our basic position is that nation building is an ongoing, always unfinished undertaking involving various practices of statecraft (which often feature the cultivation and circulation of a set of identity categories), see Gellner and Smith (1996).

4 See Teulieres (2007, p. 43) who observes “like a mirror, the figure of the migrant unmasks the collective identities and symbolic boundaries of each community.”

5 English translation of the Annex to the contract between SE and the Estonian Ministry of Culture. Both the Estonian original and the English translation are on file with the authors.

6
http://pub.stat.ee/px-web.2001/I_Databas/Multidomain_statistics/Integration/Integration.asp (accessed 4 May 2017).

7
http://pub.stat.ee/px-web.2001/I_Databas/Multidomain_statistics/Integration/PO_021.htm (accessed 4 May 2017).

8 In this context, it is important to note that not all countries in the world recognised the geopolitical borders of the Soviet Union as they did not accept the de facto annexation of the Baltic states as legal. In particular, Western countries, most notably the United States, thus supported the narrative of a continued de jure existence of the Estonian nation state.

9 Article 8 of the constitution of the Republic of Estonia stipulates “[e]very child of whose parents one is an Estonian citizen has the right to Estonian citizenship by birth” (*Constitution of the Republic of Estonia. English Translation*, 1992).

10 In general, statistical categorisations along ethnic lines can be used for integration monitoring, exclusion, and control and to document and counter discrimination (Loveman, 2014). The absence of affirmative action policies in Estonia suggests, however, in conjunction with the introduction of the “third generation” category, that the latter primarily serves as a monitoring tool for integration policies. These policies are based on a socioeconomic understanding of integration emphasising individual responsibility, in particular by requiring command of Estonian as a measure to improve the economic situation of the Russian‐speaking minority (cf. Cianetti, 2015). The introduction of the third generation category was pushed for by demographers of the University of Tallinn, who called for an unplanned meeting of the Scientific Council after violent clashes between the police and members of the Russian speaking minority in April 2007. The demographers successfully lobbied for the introduction of the third generation category to produce more fine‐grained knowledge about the Russian‐speaking minority (interview Scientific Council, May 2015; interview Statistics Estonia, May 2015). Jääts (2014) notes that statistical authorities and decision makers displayed a similar appetite for a traditional Estonian nationalism in the preparation of the PHC 2011 in their refusal to include questions on sub‐national ethnic identities in the census questionnaire.

11 The development of lists of officially recognised nationalities and nationality definitions were contested processes in which statisticians, geographers, ethnographers, government officials, and lobbyists of ethnic groups were involved. Eventually, the determination of nationality through subjective self‐definition was chosen to translate the promise of national self‐determination—one of the main factors for the military successes of the Bolsheviks during the Russian Revolution—to the individual level (cf. Hirsch, 1997).

12 This complex taxonomy of identity categories used in Estonian population statistics is indicative of the politics of belonging at work in the Estonian case. In Estonian population statistics it is, for instance, possible to create tabulations that feature members of the “third generation of the foreign‐origin population” who do not hold legal citizenship as they are of “undetermined citizenship,” whose mother tongue is Russian but who nevertheless identify as Estonian when it comes to ethnic nationality. Conversely, there are members of the “second generation of the foreign‐origin population” who do not hold Estonian citizenship, whose mother tongue is Russian, and who also identify as Russian when it comes to ethnic nationality, and so forth.

13 The Caribbean Netherlands are also referred to as the BES (Bonaire, St Eustatius, Saba) islands. Following the dominant terminology of our SN research participants, we use the former. Furthermore, we follow legal and governmental terminology in referring to the self‐governing constituent territories of the Kingdom of the Netherlands as “countries,” instead of “(nation) states” (Charter of the Kingdom of the Netherlands 1954, 17 November 2011, Article 5.1).

14 For simplicity, we have omitted two events from this table. In 1986, Aruba gained status aparte and became a country in the Kingdom. In 2010, Curaçao and St Maarten gained status aparte.

15 We describe the nature of minority policies since the 1990s focussing on “cultural integration.” On the history and consequences of Dutch integration policies and discourses, see, for instance Essed (2002); Uitermark (2012); Van Reekum and Duyvendak (2012).

16 See Statline, “Key Population Figures”: https://opendata.cbs.nl/statline/#/CBS/nl/dataset/37296ned/table?ts = 1558202225005, visited 18 May 2019.

17 We reviewed demographic publications and articles published between 2010 and 2018, available on the online SN archive (https://www.cbs.nl/nl-nl/onze-diensten/archief/archief-van-artikelen/themas/bevolking, visited on 14 August 2018). Publications specifically about the Caribbean Netherlands origin groups concern urban residence, life expectancy, teenage motherhood, and single motherhood. We also checked a broader group of publications about relationships, fertility, and family regardless of origin group. Here we found that of the 53 reviewed articles in this group, eight distinguish the Caribbean origin categories, of which four highlight teenage motherhood, and two single parenthood.

18 For other examples, see CBS (2016b).
